# Efficient Electrochemical *N*-Alkylation of *N*-Boc-Protected 4-Aminopyridines: Towards New Biologically Active Compounds

**DOI:** 10.1155/2014/621592

**Published:** 2014-03-05

**Authors:** Marta Feroci, Isabella Chiarotto, Gianpiero Forte, Giovanna Simonetti, Felicia Diodata D'Auria, Louis Maes, Daniela De Vita, Luigi Scipione, Laura Friggeri, Roberto Di Santo, Silvano Tortorella

**Affiliations:** ^1^Department of Basic and Applied Sciences for Engineering, Sapienza University of Rome, Via Castro Laurenziano 7, 00161 Rome, Italy; ^2^Department of Public Health and Infectious Diseases, Sapienza University of Rome, Piazzale Aldo Moro 5, 00185 Rome, Italy; ^3^Laboratory for Microbiology, Parasitology and Hygiene (LMPH), Faculty of Pharmaceutical, Biomedical and Veterinary Sciences, Antwerp University, 2610 Antwerp, Belgium; ^4^“Istituto Pasteur-Fondazione Cenci Bolognetti”, Department of “Chimica e Tecnologie del Farmaco”, Sapienza University of Rome, Piazzale Aldo Moro 5, 00185 Rome, Italy

## Abstract

The use of electrogenerated acetonitrile anion allows the alkylation of *N*-Boc-4-aminopyridine in very high yields, under mild conditions and without by-products. The high reactivity of this base is due to its large tetraethylammonium counterion, which leaves the acetonitrile anion “naked.” The deprotection of the obtained compounds led to high yields in *N*-alkylated 4-aminopyridines. Nonsymmetrically dialkylated 4-aminopyridines were obtained by subsequent reaction of monoalkylated ones with *t*-BuOK and alkyl halides, while symmetrically dialkylated 4-aminopyridines were obtained by direct reaction of 4-aminopyridine with an excess of *t*-BuOK and alkyl halides. Some mono- and dialkyl-4-aminopyridines were selected to evaluate antifungal and antiprotozoal activity; the dialkylated 4-aminopyridines **3ac**, **3ae** and **3ff** showed antifungal towards *Cryptococcus neoformans*; whereas **3cc**, **3ee** and **3ff** showed antiprotozoal activity towards *Leishmania infantum* and *Plasmodium falciparum*.

## 1. Introduction

*N*-Alkylated 4-aminopyridine is a common moiety in biologically active molecules. It is present, in fact, in compounds with different activities such as inhibitors of p38*α* MAP kinase [1], inhibitors of HIV-EP1 cellular transcription factor [2], inhibitors of coagulation Factor Xa [3], and *β*-chemokine receptor CCR5 antagonists in anti-HIV therapy [4]; in particular we have focused our work on the development of new CYP51 inhibitors, active both on fungal strains [5] and *Trypanosoma Cruzi *[6]. Many literature data evidenced that the pyridine group can efficaciously replace the heme-iron chelating azole moiety present in classical azole CYP51 inhibitors and, therefore, the alkylation of 4-aminopyridine (4AP) represents an important goal in organic synthesis to develop novel classes of antifungal and antiparasitic drugs [7, 8].

Due to the wide presence of these products, the alkylation of 4-aminopyridine (4AP) is therefore an important goal in organic synthesis.

Different approaches to obtain *N*-alkylated 4-aminopyridines have been reported in the literature. Some examples are the efficient condensation of 4AP with alcohols catalyzed by benzaldehyde [9] or copper [10, 11] or magnetite [12], the reaction of 4AP with an acyl chloride, and the following reduction of the amide with LiAlH_4_ [13].

**Scheme 1:**
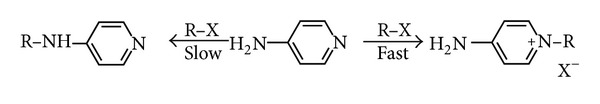
Reaction of 4-aminopyridine with alkyl halides.

The most straightforward method, however, is the direct alkylation of 4AP with alkyl halides, although it suffers from some drawbacks. The two different nitrogen atoms compete in the alkylation reaction and usually the more nucleophilic pyridine nitrogen atom reacts faster, leading to the corresponding pyridinium salt (Scheme 1) [14, 15].

In these case, the use of a very strong base is therefore necessary: *n*-BuLi was successfully used by Singh and coworkers [16], obtaining *N*-methyl- and *N*-ethyl-4-aminopyridines in 74–80% yields.

A viable alternative is the enhancement of the nucleophilicity of the amine nitrogen atom (versus the pyridine one), allowing the use of weaker bases. An example is the activation of 2-aminopyridine as formyl or Boc derivative at the amine nitrogen atom [17], with subsequent deprotonation using sodium hydride, alkylation, and deprotection with trifluoroacetic acid. The deprotonation of *N*-Boc-2-aminopyridine with NaH needs a careful control of the temperature (0–5°C) and is carried out in anhydrous DMF, with a vigorous stirring required to keep the suspension fluid.

**Scheme 2:**
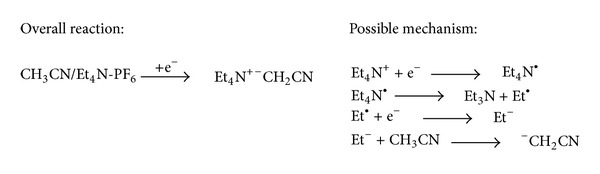
Electrogeneration of acetonitrile anion.

In this context, we envisaged the possibility to alkylate *N*-Boc-4-aminopyridine (*N*-Boc-4AP) using milder reaction conditions, that is, using electrogenerated tetraethylammonium cyanomethanide (Et4N^+-^CH_2_CN) [18]. This base, the acetonitrile anion, can be easily obtained by cathodic galvanostatic reduction of a solution of acetonitrile containing tetraethylammonium hexafluorophosphate as supporting electrolyte (Scheme 2), without by-products (the reagent is the electron), and it was successfully used by us in a good variety of reactions [19], such as the selective *N*-alkylation of bifunctional compounds [20], the Gewald reaction [21], the synthesis of *β*-lactams [22], and the synthesis of carbamates [23]. The actual mechanism for the formation of acetonitrile anion is not known, but a hypothesis based on the direct reduction of the tetraethylammonium cation has been reported (Scheme 2) [24].

The high reactivity of this base is ascribable to the large tetraethylammonium counterion, which renders the acetonitrile anion extremely reactive. Moreover, its reaction as a base gives no by-products, as the protonation restores the molecule of solvent.

## 2. Results and Discussion

**Scheme 3:**
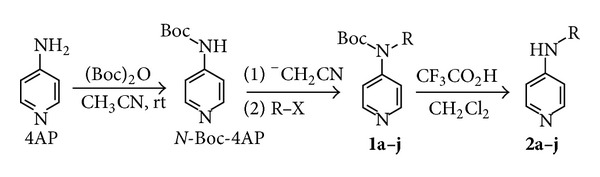
Synthesis of *N*-alkyl-4-aminopyridine.

The reaction of electrogenerated acetonitrile anion with 4AP, followed by an alkyl halide, leads to poor yields in desired compound with the pyridinium salt being the major product. This prevents the direct use of ^−^CH_2_CN with 4AP. On the other hand, if the amine nitrogen is activated as Boc derivative (*N*-Boc-4AP), the deprotonation/alkylation reaction using acetonitrile anion leads to products**1** in very high yields (Scheme 3 and Table 1, entries 1–6). The classic deprotection with trifluoroacetic acid allows obtaining the desired products**2** (Scheme 3 and Table 1, entries 1–6).

The data in Table 1 highlight that the reaction of deprotonation of *N*-Boc-4AP using electrogenerated acetonitrile anion, alkylation with both alkyl and benzyl halides, and deprotection with trifluoroacetic acid is very efficient, with overall yields of 78–86%. However, when the alkylating agent is a bromoacetophenone, the yields in alkylated product are lower and in most cases the deprotection reaction leads to the dealkylation of the starting material (Table 1, entries 7–10).

As many biologically active compounds contain the dialkylated 4-aminopyridine moiety, we tried to carry out a second alkylation on products **2a–j** using acetonitrile anion but, as expected, the high nucleophilicity of the pyridine nitrogen led to the synthesis of the corresponding pyridinium salt.

**Scheme 4:**
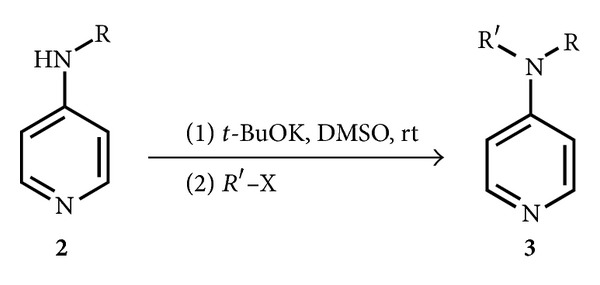
Alkylation of 4-alkylaminopyridine.

We thus carried out this second alkylation using strong bases, the most efficient being* t*-BuOK in DMSO (Scheme 4), although the yields in dialkylated 4AP were not very high. The results of this reaction are reported in Table 2.

In order to obtain symmetrically dialkylated 4AP, 4AP was subjected to deprotonation with *t*-BuOK in DMSO, adding an excess of alkylating agent. The reaction led to a mixture of mono- and dialkylated 4-aminopyridines, in moderate to acceptable yields. The results are reported in Table 3.

## 3. Biological Activity

A selection of synthesized compounds was *in vitro* tested to evaluate antifungal activity against different strains of *C. albicans*, *C. parapsilosis,* and *Cryptococcus neoformans*; data are reported in Table 4. As can be evidenced the nonsymmetrical dialkylated 4APs **3ac** and **3ae** showed a moderate antifungal activity towards *C. albicans* and *C. parapsilosis* with MIC_50_ values of 32 *μ*g/mL and showed an interesting activity against *Cryptococcus neoformans*, with MIC_50_ values of 0.4 and 4 *μ*g/mL, respectively. Otherwise, the symmetrical dialkylated 4APs **3cc**, **3ee** and the Boc-protected monoalkylated 4APs **1b**, **1e**, **1f** showed poor antifungal activity with MIC_50_ and MIC_100_ ≥ 64 *μ*g/mL.

Furthermore, the symmetrical dialkylated 4APs **3cc**, **3ee,** and **3ff** were *in vitro* tested to evaluate the activity against *Trypanosoma cruzi*, *Trypanosoma brucei*, *Leishmania infantum,* and *Plasmodium falciparum*; the results are summarized in Table 5.

As can be evidenced, all tested compounds showed a moderate activity versus *P. falciparum *and an interesting activity towards *L. infantum* with IC_50_ values lower than the reference drug miltefosine; otherwise, they resulted scarcely active against *T. cruzi* and *T. brucei*. Moreover, these compounds also showed low toxic activity versus growing MRC-5 cells.

## 4. Conclusion

In conclusion, we demonstrated the usefulness of electrogenerated acetonitrile anion in the alkylation of *N*-Boc 4-aminopyridines, both from the point of view of the high yields and of the cleanliness of the reaction (no by-products). The deprotection of *N*-Boc 4-aminopyridines allowed obtaining monoalkylated 4-aminopyridine in very high yields. The following alkylation, by means of *t*-BuOK and alkyl halides, led to nonsymmetrically dialkylated 4-aminopyridine, while symmetrically dialkylated products were obtained directly from 4-aminopyridine by reaction with an excess of *t*-BuOK and alkyl halide.

Furthermore, it can also be concluded that the monoalkylation of the 4AP leads to inactive products and otherwise interesting activity against fungi and some protozoa can be obtained by dual, symmetrical, or nonsymmetrical dialkylation of the amino group of 4AP; these active molecules can be considered as lead compound to develop new antifungal and antiprotozoal compounds.

## 5. Materials and Methods

### 5.1. General

Acetonitrile was distilled twice from P_2_O_5_ and CaH_2_. Commercially available reagents were used without further purification. The Boc protection of 4-aminopyridine was carried out following the literature [25].

*4-[N-(tert-Butoxycarbonyl)amino]pyridine N-Boc-4AP.* To a solution of di-*tert*-butyl dicarbonate (3 mmol) in acetonitrile (3 cm^3^) at room temperature 4-aminopyridine (3 mmol) was slowly added. This mixture was then allowed to stir for 3 h at room temperature. The solvent was evaporated and the crude 4-[*N*-(*tert*-butoxycarbonyl)amino]pyridine (>95%) was used in the electrolyses without further purification. R_f_ (30% ethyl acetate in light petroleum ether) 0.20; ^1^H NMR (200 MHz, CDCl_3_) *δ* 1.53 (s,9H), 6.9 (bs, 1H), 7.32 (dd,  *J* = 4.8, 1.6 Hz, 2H), 8.45 (dd,  *J* = 4.8, 1.6 Hz, 2H); ^13^C NMR (50 MHz, CDCl_3_) *δ* 28.2, 81.7, 112.3, 145.6, 150.3, 151.9; EIMS, *m/z*: 194 (M^.+^, 1%), 137 (2%), 121 (5%), 120 (8%), 94 (50%), 78 (4%), 57 (100%).

### 5.2. Electrochemical ALkylation of N-Boc-4AP

Constant current electrolyses (*I* = 25 mA cm^−2^) were performed under a nitrogen atmosphere, at 20°C, using an Amel Model 552 potentiostat equipped with an Amel Model 731 integrator. All the experiments were carried out in a divided glass cell separated through a porous glass plug filled up with a layer of gel (i.e., methyl cellulose 0.5% volume dissolved in DMF-Et_4_NPF_6_ 1.0 mol dm^−3^); Pt spirals (apparent areas 0.8 cm^2^) were used both as cathode and anode. MeCN-Et_4_NPF_6_ 0.1 mol dm^−3^ was used as solvent-supporting electrolyte system (catholyte: 20 cm^3^; anolyte: 5 cm^3^). 1 mmol of *N*-Boc-4-aminopyridine was present in the catholyte. After 145 C were passed, the current was switched off and 1 mmol of alkylating agent was added to the catholyte. The solution was kept under stirring at room temperature for 2 hours; then the solvent was evaporated under reduced pressure and the residue was purified by flash column chromatography, using a mixture of ethyl acetate/light petroleum ether 2/8 in volume, obtaining the pure products.

Flash column chromatography was carried out using Merck 60 kieselgel (230–400 mesh) under pressure. GC-MS measurements were carried out on SE 54 capillary column using a Fisons 8000 gas chromatograph coupled with a Fisons MD 800 quadrupole mass selective detector. ^1^H and ^13^C NMR spectra were recorded at room temperature using a Bruker AC 200 spectrometer using CDCl_3_ as internal standard.

*tert-Butyl (octyl)pyridin-4-ylcarbamate* **1a**. R_f_ (80% ethyl acetate in dichloromethane) 0.60; ^1^H NMR (200 MHz, CDCl_3_) *δ* 0.88 (t,   *J* = 6.5 Hz, 3H), 1.20–1.30 (m, 10H), 1.49–1.86 (m, 3H), 1.50 (s, 9H), 3.69 (app  t, *J* = 7.6 Hz, 2H), 7.24 (dd,  *J* = 6.2, 1.6 Hz, 2H), 8.51 (dd,  *J* = 6.2, 1.6 Hz, 2H); ^13^C NMR (50 MHz, CDCl_3_) *δ* 14.0, 22.6, 26.7, 28.2, 28.4, 29.1, 31.7, 48.7, 81.4, 118.8, 150.0, 150.1, 153.4.

*tert-Butyl (3-phenylpropyl)pyridin-4-ylcarbamate* **1b**. R_f_ (50% ethyl acetate in light petroleum ether) 0.46; ^1^H NMR (200 MHz, CDCl_3_) *δ* 1.48 (s, 9H), 1.86–2.02 (m, 2H), 2.64 (t, *J* = 7.6 Hz, 2H), 3.74 (app  t,   *J* = 7.6 Hz, 2H), 7.12–7.33 (m, 7H), 8.49 (dd, *J* = 4.8, 1.6 Hz, 2H); ^13^C NMR (50 MHz, CDCl_3_) *δ* 28.2, 30.0, 33.0, 48.3, 81.6, 118.9, 126.1, 128.3, 128.5, 141.0, 149.7, 150.3, 153.4; EIMS, *m/z*: M^.+^ absent, 212 (5%), 107 (100%), 105 (5%), 91 (25%), 78 (27%), 77 (12%).

*tert-Butyl (benzyl)pyridin-4-ylcarbamate* **1c**. R_f_ (60% ethyl acetate in light petroleum ether) 0.58; ^1^H NMR (200 MHz, CDCl_3_) *δ* 1.45 (s, 9H), 4.94 (s, 2H), 7.20–7.37 (m, 7H), 8.46 (dd,  *J* = 4.8, 1.6 Hz, 2H); ^13^C NMR (50 MHz, CDCl_3_) *δ* 28.1, 52.5, 82.1, 118.2, 126.3, 127.3, 128.7, 137.7, 150.2, 150.1, 153.5; EIMS, *m/z*: M^.+^ absent, 227 (4%), 183 (14%), 91 (100%), 78 (7%), 57 (51%).

*tert-Butyl (2,6-dichlorobenzyl)pyridin-4-ylcarbamate* **1d**. R_f_ (50% ethyl acetate in light petroleum ether) 0.60; ^1^H NMR (200 MHz, CDCl_3_) *δ* 1.48 (s, 9H), 5.29 (s, 2H), 7.02–7.21 (m, 5H), 8.41 (dd,   *J* = 4.8, 1.6 Hz, 2H); ^13^C NMR (50 MHz, CDCl_3_) *δ* 28.2, 46.7, 81.7, 121.5, 128.6, 129.5, 131.7, 136.1, 148.1, 149.9, 153.3; EIMS, *m/z*: 352 (M^.+^, 1%), 252 (3%), 163 (6%), 161 (30%), 159 (42%), 78 (76%), 51 (100%).

*tert-Butyl (4-fluorobenzyl)pyridin-4-ylcarbamate* **1e**. R_f_ (50% ethyl acetate in light petroleum ether) 0.49; ^1^H NMR (200 MHz, CDCl_3_) *δ* 1.45 (s, 9H), 4.89 (s, 2H), 6.97–7.22 (m, 6H), 8.47 (app d, *J* = 6.0 Hz, 2H); ^13^C NMR (50 MHz, CDCl_3_) *δ* 28.1, 51.8, 82.2, 116.6 (d,  *J* = 21.5 Hz), 118.4, 128.1 (d, *J* = 8.0 Hz), 133.4 (d,  *J* = 3.2 Hz), 149.9, 150.3, 153.4, 162.2 (d,*J* = 245.4 Hz); EIMS, *m/z*: 302 (M^.+^, 1%), 245 (4%), 201 (53%), 108 (100%), 78 (42%), 57 (100%).

*tert-Butyl (4-trifluoromethylbenzyl)pyridin-4-ylcarbamate* **1f**. R_f_ (50% ethyl acetate in light petroleum ether) 0.41; ^1^H NMR (200 MHz, CDCl_3_) *δ* 1.46 (s, 9H), 4.99 (s, 2H), 7.20–7.36 (m, 4H), 7.61 (app d,  *J* = 8.4 Hz, 2H), 8.49 (app d, *J* = 6.2 Hz, 2H); ^13^C NMR (50 MHz, CDCl_3_) *δ* 28.1, 52.2, 82.5, 118.1, 124.0 (q,  *J* = 271.9 Hz), 125.7 (q,  *J* = 3.7 Hz), 126.6, 129.7 (q,  *J* = 32.3 Hz), 141.9, 149.8, 150.4, 153.3; EIMS, *m/z*: M^.+^ absent, 251 (9%), 158 (34%), 145 (2%), 78 (25%), 69 (9%), 57 (100%).

*tert-Butyl (2-oxo-2-phenylethyl)pyridin-4-ylcarbamate* **1 g**. R_f_ (60% ethyl acetate in light petroleum ether) 0.50; ^1^H NMR (200 MHz, CDCl_3_) *δ* 1.46 (s,  9H), 5.09 (s, 2H), 7.26 (d,  *J* = 6.4 Hz, 2H), 7.48–7.68 (m, 3H), 7.99 (d,  *J* = 8.2 Hz, 2H), 8.47–8.55 (m, 2H); ^13^C NMR (50 MHz, CDCl_3_) *δ* 28.1, 55.5, 82.5, 118.7, 127.9, 128.9, 133.9, 134.7, 150.2, 153.2, 193.7.

*tert-Butyl (2-(4-fluoropheny)-2oxoethyl)pyridin-4-ylcarbamate* **1 h**. R_f_ (50% ethyl acetate in light petroleum ether) 0.50; ^1^H NMR (200 MHz, CDCl_3_) *δ* 1.46 (s, 9H), 5.05 (s, 2H), 7.15–7.27 (m, 4H), 7.99–8.06 (m, 2H), 8.52 (dd, *J* = 5.0, 1.4 Hz, 2H); ^13^C NMR (50 MHz, CDCl_3_) *δ* 28.1, 55.4, 82.6, 116.2 (d,  *J* = 22.0 Hz), 118.8, 130.6 (d,  *J* = 9.4 Hz), 131.1 (d,  *J* = 3.2 Hz), 150.1, 150.2, 153.2, 166.2 (d,  *J* = 256.1 Hz), 192.2.

*tert-Butyl (2-(4-chloropheny)-2oxoethyl)pyridin-4-ylcarbamate* **1i**. R_f_ (20% ethyl acetate in dichloromethane) 0.30; ^1^H NMR (200 MHz, CDCl_3_) *δ* 1.45 (s, 9H), 5.04 (s, 2H), 7.21 (dd,  *J* = 4.6, 1.6 Hz, 2H), 7.50 (d,  *J* = 8.8 Hz, 2H), 7.93 (d,  *J* = 8.0 Hz, 2H), 8.51 (dd,  *J* = 4.6, 1.6 Hz, 2H); ^13^C NMR (50 MHz, CDCl_3_) *δ* 28.1, 55.4, 82.6, 118.8, 129.3, 133.0, 140.5, 149.9, 150.3, 153.1, 192.7.

*tert-Butyl (2-(4-methoxypheny)-2oxoethyl)pyridin-4-ylcarbamate* **1j**. R_f_ (40% ethyl acetate in dichloromethane) 0.50; ^1^H NMR (200 MHz, CDCl_3_) *δ* 1.46 (s, 9H), 3.90 (s, 3H), 5.04 (s, 2H), 6.99 (d,  *J* = 9.0 Hz, 2H), 7.24 (dd,  *J* = 4.8, 1.6 Hz, 2H), 7.97 (d,  *J* = 9.0 Hz, 2H), 8.50 (dd,  *J* = 4.8, 1.6 Hz, 2H); ^13^C NMR (50 MHz, CDCl_3_) *δ* 28.1, 55.1, 55.5, 82.3, 114.1, 118.7, 127.8, 130.2, 150.2, 153.3, 164.1, 192.0.

### 5.3. Deprotection of Compounds **1a–j**

To a solution of **1** (1 mmol) in CH_2_Cl_2_ (5 cm^3^), kept at 0°C, 1 cm^3^ of CF_3_COOH was added. This mixture was allowed to stir for 3 h at 0°C. The solution was then mixed with aqueous sodium carbonate till pH 8 and extracted with ethyl acetate. The solvent was removed under reduced pressure and the mixture was purified by flash chromatography, yielding pure compound **2**.

*N-(Octyl)pyridin-4-amine* **2a**. R_f_ (20% dichloromethane in ethyl acetate) 0.16; ^1^H NMR (200 MHz, CDCl_3_) *δ* 0.89 (t,  *J* = 7.2 Hz, 3H), 1.25–1.34 (6H), 1.61–1.70 (m, 2H), 2.89–3.12 (m, 2H), 3.15–3.24 (m, 2H), 5.43–5.48 (m, 2H), 6.57 (d,  *J* = 5.2 Hz, 2H), 8.11 (d,  *J* = 5.2 Hz, 2H); ^13^C NMR (50 MHz, CDCl_3_) *δ* 14.1, 22.6, 27.0, 28.8, 29.2, 29.2, 31.8, 42.9, 107.4, 155.2.

*N-(3-Phenylpropyl)pyridin-4-amine* **2b**. R_f_ (ethyl acetate) 0.46; ^1^H NMR (200 MHz, CD_3_CN) *δ* 1.91–2.06 (m, 2H), 2.71 (t,  *J* = 7.4 Hz, 2H), 3.17–3.27 (m, 2H), 4.9 (bs, 1H), 6.62–6.66 (m, 2H), 7.14–7.46 (m, 5H), 7.45–7.97 (m, 2H); ^13^C NMR (50 MHz, CD_3_CN) *δ* 29.8, 32.5, 42.0, 107.3, 125.9, 128.3, 128.4, 141.0, 141.5, 157.8; EIMS, *m/z*: 212 (M^.+^, 1%), 107 (100%), 91 (43%), 78 (13%).

*N-(Benzyl)pyridin-4-amine* **2c**. R_f_ (ethyl acetate) 0.57; ^1^H NMR (200 MHz, CDCl_3_) *δ* 4.46 (d,  *J* = 6.0 Hz, 2H), 6.7 (bs, 2H), 7.1 (bs, 1H), 7.21–7.41 (m, 5H), 8.0 (bs, 2H); ^13^C NMR (50 MHz, CDCl_3_) *δ* 46.8, 107.4, 127.2, 127.6, 128.8, 137.7, 148.6, 154.0; EIMS, *m/z*: 184 (M^.+^, 15%), 183 (15%), 107 (5%), 91 (100%), 78 (16%).

*N-(2,6-Dichlorobenzyl)pyridin-4-amine* **2d**. R_f_ (ethyl acetate) 0.38; ^1^H NMR (200 MHz, CD_3_CN) *δ*4.59 (s, 2H), 6.0 (bs, 1H), 6.68 (dd,  *J* = 5.2, 1.4 Hz, 2H), 7.27–7.47 (m, 3H), 8.41 (app d,  *J* = 5.4   Hz, 2H); ^13^C NMR (50 MHz, CD_3_CN) *δ* 42.2, 107.4, 128.7, 130.4, 132.8, 136.0, 147.3, 154.6; EIMS, *m/z*: 256 (M^.+^ + 4, 1%), 254 (M^.+^ + 2, 7%), 252 (M^.+^, 14%), 162 (10%), 160 (67%), 158 (100%), 78 (37%).

*N-(4-Fluorobenzyl)pyridin-4-amine* **2e**. R_f_ (ethyl acetate) 0.27; ^1^H NMR (200 MHz, CDCl_3_) *δ* 4.38 (d, *J* = 5.4 Hz, 2H), 5.1 (bs, 1H), 6.5 (bs, 2H), 7.02–7.10 (m, 2H), 7.30–7.34 (m, 2H), 8.2 (bs, 2H); ^13^C NMR (50 MHz, CDCl_3_) *δ* 46.3, 107.8, 115.8 (d,  *J* = 21.5 Hz), 129.0 (d,  *J* = 8.1 Hz), 133.1, 148.5, 153.8, 166.8 (d,  *J* = 205.9 Hz),; EIMS, *m/z*: 202 (M^.+^, 5%), 107 (16%), 109 (100%), 78 (16%).

*N-(4-Trifluoromethylbenzyl)pyridin-4-amine* **2f**. R_f_ (ethyl acetate) 0.25; ^1^H NMR (200 MHz, CDCl_3_) *δ* 4.51 (d,   *J* = 6.0 Hz, 2H), 6.2 (bs, 1H), 6.6 (bs, 2H), 7.54 (d,  *J* = 8.0 Hz, 2H), 7.67 (d,  *J* = 8.0 Hz, 2H), 8.1 (bs, 2H); ^13^C NMR (50 MHz, CDCl_3_) *δ* 45.4, 107.8, 124.4 (q,  *J* = 271.0 Hz), 125.4 (q,  *J* = 3.9 Hz), 128.4 (q, *J* = 31.9 Hz), 127.7, 143.2, 146.7, 155.0; EIMS, *m/z*: 252 (M^.+^, 80%), 183 (11%), 159 (100%), 107 (52%), 78 (31%).

*1-(4-Methoxyphenyl)-2-(pyridin-4-ylamine)ethan-1-one* **2 g**. R_f_ (50% ethyl acetate in ethanol) 0.15; ^1^H NMR (200 MHz, CDCl_3_) *δ* 3.90 (s, 3H), 4.57 (d,  *J* = 3.8 Hz, 2H), 4.6 (bs, 1H), 6.58 (d, *J* = 6.2 Hz, 2H), 6.98 (d,  *J* = 9.0 Hz, 2H), 7.98 (d,  *J* = 9.0 Hz, 2H), 8.19 (d,  *J* = 6.2 Hz, 2H); ^13^C NMR (50 MHz, CDCl_3_) *δ* 48.2, 55.6, 108.1, 114.1, 127.2, 130.2, 150.8, 153.6, 164.5, 191.6.

### 5.4. Alkylation of Compounds **2a,c,e**

To a solution of **2** (1 mmol) in anhydrous DMSO (2 cm^3^), kept at rt under N_2_, 1.5 mmol of *t*-BuOK was added. This mixture was allowed to stir for 20 min at rt; then 1 mmol of alkyl halide was added and the solution was kept under stirring at rt for 4 h. The solution was then mixed with water and extracted with dichloromethane. The solvent was removed under reduced pressure and the mixture was purified by flash chromatography, yielding pure compound **3**.

*N-Benzyl-N-octylpyridin-4-amine* **3ac** *. *R_f_ (80% ethyl acetate in ethanol) 0.38; ^1^H NMR (200 MHz, CDCl_3_) *δ* 0.86–0.92 (m, 3H), 1.20–1.40 (m, 10H), 1.63–1.70 (m, 2H), 3.42 (app t,  *J* = 7.6 Hz, 2H), 4.59 (s, 2H), 6.51 (d,  *J* = 5.2 Hz, 2H), 7.14–7.38 (m, 5H), 8.18 (d,  *J* = 5.2  Hz, 2H); ^13^C NMR (50 MHz, CDCl_3_) *δ* 14.1, 22.6, 26.9, 27.0, 29.2, 29.4, 29.7, 31.8, 50.7, 53.4, 106.9, 126.2, 127.3, 128.8, 136.8, 148.9, 153.6.

*N-(4-Fluorobenzyl)-N-octylpyridin-4-amine* **3ae**. R_f_ (ethyl acetate) 0.40; ^1^H NMR (200 MHz, CDCl_3_) *δ* 0.86–0.92 (m, 3H), 1.23–1.35 (m, 10H), 1.62–1.72 (m, 2H), 3.42 (app t,  *J* = 7.8 Hz, 2H), 4.58 (s, 2H), 6.56 (app d,  *J* = 6.4 Hz, 2H), 6.99–7.16 (m, 4H), 8.17 (app d,  *J* = 6.4 Hz, 2H); ^13^C NMR (50 MHz, CDCl_3_) *δ* 14.1, 19.2, 22.6, 26.9, 29.2, 29.3, 31.7, 51.3, 53.3, 107.4, 116.1 (d, *J* = 21.7 Hz), 127.9 (d,  *J* = 8.1 Hz), 130.9 (d,  *J* = 3.5 Hz), 144.9, 155.3, 162.3 (d,  *J* = 246.5 Hz).

*N-(4-Fluorobenzyl)-N-(3-phenylpropyl)pyridin-4-amine* **3be**. R_f_ (ethyl acetate: *n*-hexane: methanol 50 : 33 : 17) 0.48; ^1^H NMR (200 MHz, CDCl_3_) *δ* 1.91–2.03 (m, 2H), 2.68 (t,  *J* = 7.4 Hz, 2H), 3.43 (app t, *J* = 7.8 Hz, 2H), 4.54 (s, 2H), 6.44 (app d,  *J* = 5.4 Hz, 2H), 6.96–7.36 (m, 9H), 8.15 (bs, 2H); ^13^C NMR (50 MHz, CDCl_3_) *δ* 28.1, 33.0, 49.9, 52.9, 106.8, 115.8 (d,  *J* = 21.6 Hz), 126.3, 127.9 (d,  *J* = 8.0 Hz), 128.3, 128.6, 132.1 (d,  *J* = 3.2 Hz), 140.7, 147.9, 153.7, 162.1 (d, *J* = 245.8 Hz).

### 5.5. Dialkylation of 4-Aminopyridine

To a solution of 4AP (1 mmol) in anhydrous DMSO (2 cm^3^), kept at rt under N_2_, 2 mmol of *t*-BuOK was added. This mixture was allowed to stir for 20 min at rt; then 2 mmol of alkyl halide was added and the solution was kept under stirring at rt for 4 h. The solution was then mixed with water and extracted with dichloromethane. The solvent was removed under reduced pressure and the mixture was purified by flash chromatography, yielding pure compound **3**.

*N,N-Di(3phenylpropyl)pyridin-4-amine* **3bb** *. *R_f_ (80% ethyl acetate in dichloromethane) 0.20; ^1^H NMR (200 MHz, CD_3_CN) *δ* 1.84–1.99 (m, 4H), 2.65 (t,  *J* = 7.5 Hz, 4H), 3.29 (t,  *J* = 7.5 Hz, 4H), 6.30 (d,  *J* = 5.6 Hz, 2H), 7.17–7.31 (m, 10H), 8.13 (d,  *J* = 5.6 Hz, 2H); ^13^C NMR (50 MHz, CD_3_CN) *δ* 28.3, 33.1, 49.5, 106.4, 126.2, 128.3, 128.5, 141.1, 140.7, 152.4.

*N,N-Dibenzylpyridin-4-amine* **3cc** *. *R_f_ (80% ethyl acetate in ethanol) 0.32; ^1^H NMR (200 MHz, CDCl_3_) *δ* 4.67 (s, 4H), 6.58 (dd,  *J* = 4.8, 1.6 Hz, 2H), 7.19–7.40 (m, 10H), 8.20 (dd,  *J* = 4.8, 1.6 Hz, 2H); ^13^C NMR (50 MHz, CDCl_3_) *δ* 53.2, 107.1, 126.4, 127.4, 128.9, 136.8, 150.2, 153.9.

*N,N–Di(4-fluorobenzyl)pyridin-4-amine* **3ee** *. *R_f_ (80% ethyl acetate in dichloromethane) 0.40; ^1^H NMR (200 MHz, CDCl_3_) *δ* 4.61 (s, 4H), 6.56 (dd,  *J* = 5.0, 1.6 Hz, 2H), 6.99–7.19 (m, 8H), 8.22 (dd, *J* = 5.0, 1.6 Hz, 2H); ^13^C NMR (50 MHz, CDCl_3_) *δ* 52.5, 107.1, 115.8 (d,  *J* = 21.6 Hz), 128.1 (d,  *J* = 8.0 Hz), 132.3 (d,  *J* = 3.2 Hz), 150.3, 153.6, 162.2 (d,  *J* = 245.8 Hz).

*N,N-Di(4-trifluoromethylbenzyl)pyridin-4-amine* **3ff**. R_f_ (80% ethyl acetate in dichloromethane) 0.25; ^1^H NMR (200 MHz, CDCl_3_) *δ* 4.73 (s, 4H), 6.58 (dd, *J* = 5.0, 1.6 Hz, 2H), 7.36–7.59 (m, 8H), 8.26 (dd, *J* = 5.0, 1.6 Hz, 2H); ^13^C NMR (50 MHz, CDCl_3_) *δ* 53.1, 107.1, 123.3 (q,  *J* = 3.7 Hz), 123.8 (q,  *J* = 272.3 Hz), 124.5 (q,  *J* = 3.8 Hz), 129.7, 131.4 (q,  *J* = 32.4 Hz), 137.7, 150.4, 153.4.

### 5.6. Biological Assays

#### 5.6.1. Antifungal Assay

*Organisms. *For the antifungal evaluation, strains obtained from the American Type Culture Collection (ATCC, Rockville, MD, USA), the German Collection of Microorganisms (DSMZ, Braunschweig, Germany), and the Pharmaceutical Microbiology Culture Collection (PMC, Department of Public Health and Infectious Diseases, “Sapienza” University, Rome, Italy) were tested. The strains were *Candida albicans *(ATCC 10231, ATCC 10261, ATCC 24433, ATCC 90028, 3153, PMC 1002, PMC 1011, and PMC 1030)*, C. parapsilosis *ATCC22019, *C. parapsilosis *DSM 11224*, C. tropicalis *DSM 11953, *C. tropicalis *PMC 0908*, C. tropicalis *PMC 0910, *C. glabrata *PMC 0805, *C. krusei *DSM 6128, and *C. krusei *PMC 0613*, Cryptococcus neoformans *(DSM 11959, PMC 2102, PMC 2107, PMC 2111, and PMC 2136), dermatophytes (*Trichophyton mentagrophytes *DSM 4870*, T. mentagrophytes *PMC6509*, Microsporum gypseum *DSM 7303, and *M. gypseum *PMC 7331). All of the strains were stored and grown in accordance with the procedures of the Clinical and Laboratory Standards Institute (CLSI) [26, 27].

*Antifungal Susceptibility Assays. In vitro *antifungal susceptibility was evaluated using the CLSI broth microdilution methods [26, 27]. Fluconazole and Amphotericin B were used as reference drugs. The final concentration ranged from 0.125 to 64 *μ*g/mL. The compounds were dissolved previously in DMSO at concentrations 100 times higher than the highest desired test concentration and successively diluted in test medium in accordance with the procedures of the CLSI [28]. Microdilution trays containing 100 *μ*L of serial twofold dilutions of compounds in RPMI 1640 medium (Sigma-Aldrich, St. Louis, MO, USA) were inoculated with an organism suspension adjusted to attain a final inoculum concentration of 1.0 × 10^3^–1.5 × 10^3^ cells/mL for yeasts and 0.4 × 10^4^–5 × 10^4^ CFU/mL for dermatophytes. The panels were incubated at 35°C and observed for the presence of growth at 48 h (*Candida *spp.) and 72 h (*C. neoformans *and dermatophytes).

The minimal inhibitory concentration (MIC) was, for yeasts, the lowest concentration that showed ≥ 50% growth inhibition compared with the growth control and, for dermatophytes, the lowest concentration that showed ≥ 80% growth inhibition compared with the growth control. The MIC_100_ was the lowest drug concentration that prevented 100% of growth with respect to the untreated control. According to CSI protocols, the fluconazole MIC_50_ and the amphotericin B MIC_100_ were calculated (22,23). The results were expressed as the geometric mean (G M) of the MIC values.

#### 5.6.2. Antiprotozoal Assay

For the evaluation of antiprotozoal and cytotoxic activity an integrated panel of microbial screens and standard screening methodologies were adopted as previously described [29] on the following organisms: chloroquine-resistant *P. falciparum *K 1-strain; *L. infantum *MHOM/MA (BE)/67 amastigote stage; suramin-sensitive* Trypanosoma brucei *Squib-427 strain; *Trypanosoma cruzi* Tulahuen CL2 (benznidazole-sensitive) strain; human fetal lung fibroblast cells (MRC-5 SV2).

All assays were performed in triplicate. Compounds were tested at 5 concentrations (64, 16, 4, 1, and 0.25 *μ*g/mL) to establish a full dose titration and determine the IC_50_ (inhibitory concentration 50%). The final in-test concentration of DMSO did not exceed 0.5%, which is known not to interfere with the different assays [29].

## Figures and Tables

**Table 1:** Alkylation reaction of *N*-Boc-4AP using electrogenerated acetonitrile anion in MeCN-0.1 M TEAHFP, followed by deprotection with trifluoroacetic acid^a^.

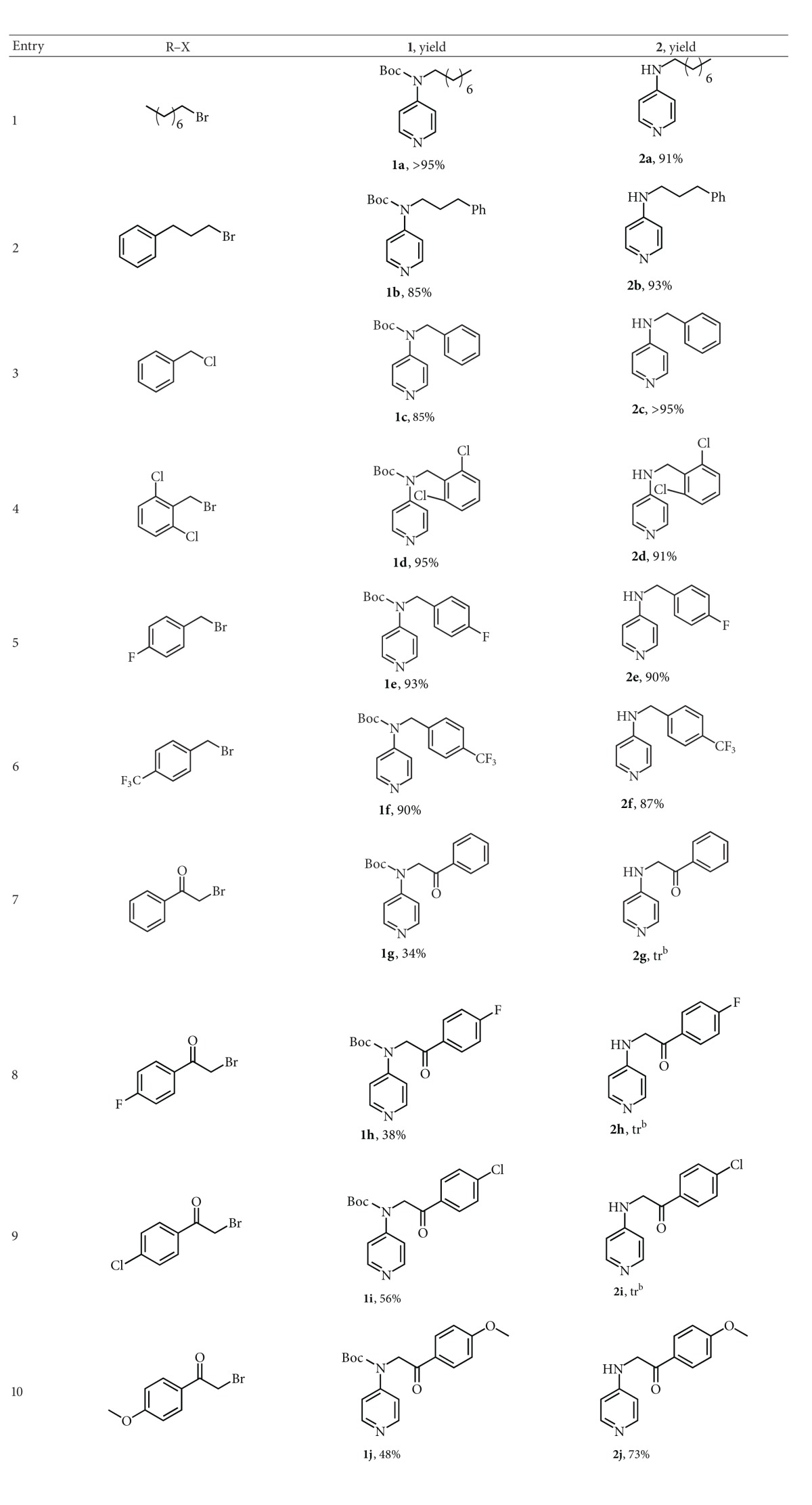

^a^The reduction was conducted under galvanostatic conditions (20 mA cm^−2^), on Pt electrodes in a divided cell at rt, on 20 mL MeCN-0.1 M TEAHFP solution containing 1 mmol of 4AP. At the end of the electrolysis, 1 mmol of alkylating agent was added. After 2 h at rt, usual workup afforded the products. Deprotection was carried out as described in the experimental part. All the yields are in isolated products.^ b^When compounds **2g–i** were subjected to deprotection with trifluoroacetic acid, a large amount of 4AP was obtained.

**Table 2:** Alkylation reaction of 4-alkylaminopyridines with *t*-BuOK in DMSO^a^.

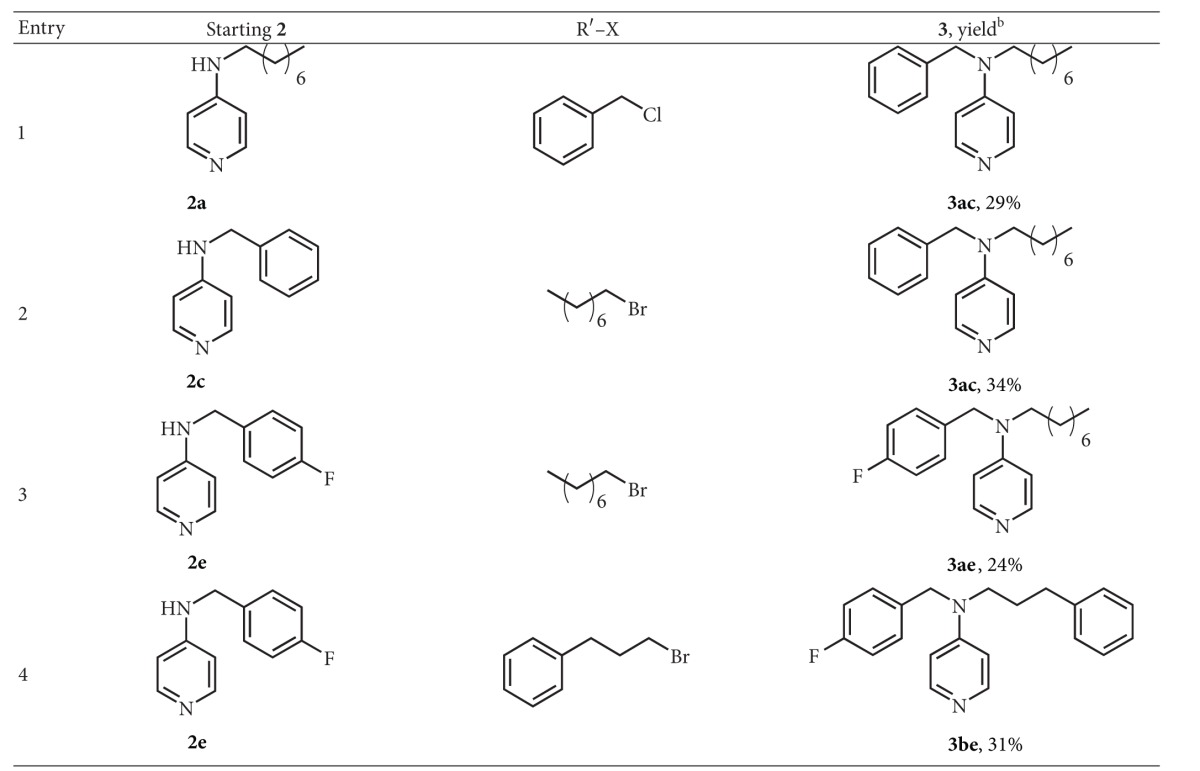

^a^1 mmol of **2** in 2 mL of anhydrous DMSO, at rt, under N_2_. Then 1.5 mmol of *t*-BuOK were added, followed by 1 mmol of halide after 20 min. The reaction was kept under stirring for 4 h. ^b^All the yields are in isolated products.

**Table 3:** Dialkylation reaction of 4-aminopyridine with *t*-BuOK in DMSO^a^.

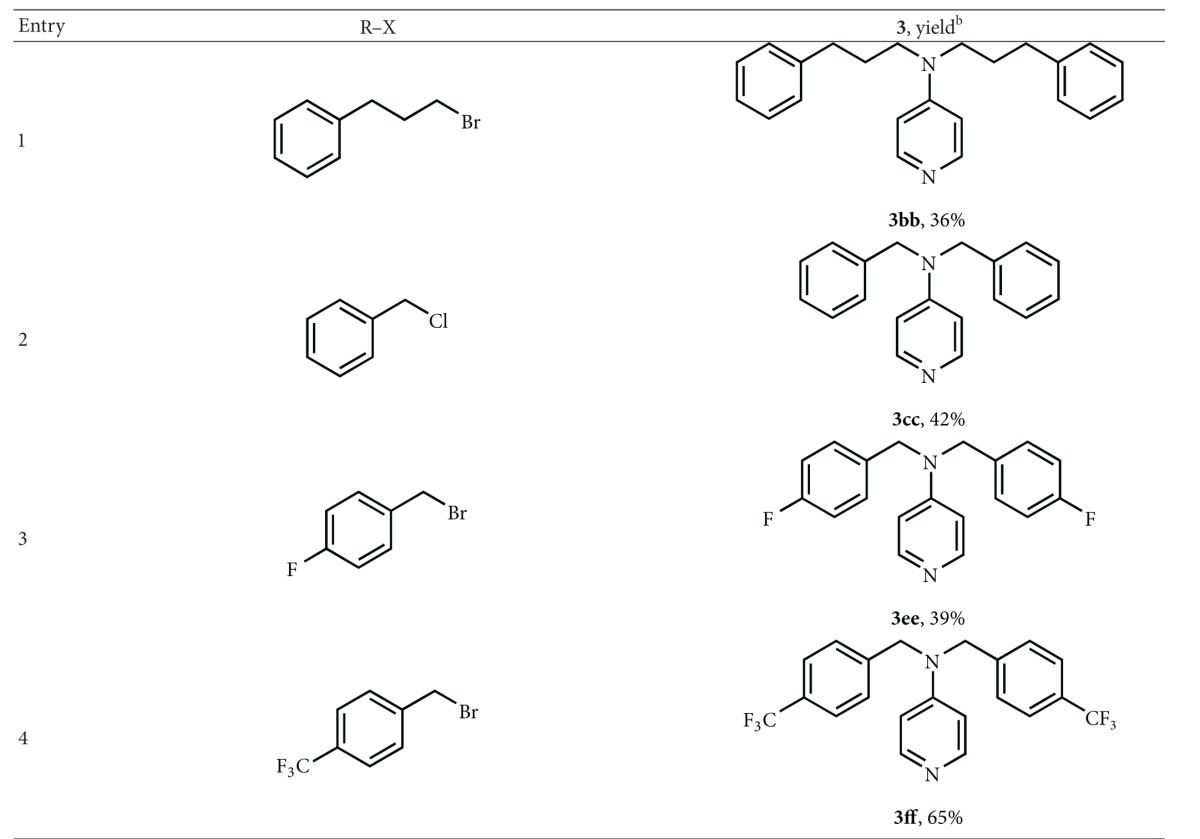

^a^1 mmol of 4AP in 2 mL of anhydrous DMSO, at rt, under N_2_. Then 2 mmol of *t*-BuOK were added, followed by 2 mmol of halide after 20 min. The reaction was kept under stirring for 4 h. ^b^All the yields are in isolated products.

**Table 4:** Antifungal activity of selected 4APs^a^.

Compound	*Candida albicans* (3 strains)		*Candida parapsilosis* ATCC22019		*Cryptococcus neoformans* (2 strains)	
	MIC_50_ (*μ*g/mL)	MIC_100_ (*μ*g/mL)	MIC_50_ (*μ*g/mL)	MIC_100_ (*μ*g/mL)	MIC_50_ (*μ*g/mL)	MIC_100_ (*μ*g/mL)
**1b**	>64	>64	>64	>64	nd	nd
**1e**	>64	>64	>64	>64	nd	nd
**1f**	>64	>64	>64	>64	nd	nd
**2e**	>64	>64	64	>64	nd	nd
**2f**	>64	>64	>64	>64	nd	nd
**3ac**	11.3^b^	26.9^b^	32	32	0.4^c^	nd
**3ae**	26.9^b^	32^b^	nd	nd	4^c^	nd
**3cc**	>64	>64	>64	>64	64	>64
**3ee**	>64	>64	>64	>64	32	64
**3ff**	>64	>64	>64	>64	8	32
Fluconazole	1	nd	2	nd	2	nd
Amphotericin B	nd	0.79	nd	1	nd	0.5

^a^The values are expressed as geometric mean of minimum inhibitory concentration (MIC) determined using Clinical and Laboratory Standard Institute (CLSI) protocol M27-A3. MIC_50_: lowest drug concentration that prevented 50% of growth with respect to the untreated control control. MIC_100_: lowest drug concentration that prevented 100% of growth with respect to the untreated control. ^b^8 *Candida  albicans* strains were tested. ^c^4 *Cryptococcus neoformans* strains were tested.

**Table 5:** Anti-parasitic activity of selected di-alkylated 4APs.

		T. cruzi^a^ IC_50_ *μ*M	T. brucei^b^ IC_50_ *μ*M	L. inf^c^ IC_50_ *μ*M	Pf-K1^d^ IC_50_ *μ*M	MRC-5 IC_50_ *μ*M
	Reference drug	Benznidazole (IC_50_ = 1.95)	Suramine (IC_50_ = 0.02)	Miltefosine (IC_50_ = 10.4)	Chloroquine (IC_50_ = 0.14)	Tamoxifen (IC_50_= 15.2)
**3cc**		20.16	31.54	8.06	1.59	39.01
**3ee**		9.57	28.23	8.64	1.87	30.09
**3ff**		2.44	6.62	2.16	1.33	7.65

^a^ *T. cruzi *Tulahuen C4 amastigote stage.^ b^ *T. brucei rhodesiense* STIB 900 trypomastigote stage. ^c^ *L. donovani* MHOM-ET-67/L82 amastigote stage. ^d^ *P.  falciparum* K1 IEF.
